# Endometrial Stromal Cells of Women with Recurrent Miscarriage Fail to Discriminate between High- and Low-Quality Human Embryos

**DOI:** 10.1371/journal.pone.0041424

**Published:** 2012-07-25

**Authors:** Charlotte H. E. Weimar, Annemieke Kavelaars, Jan J. Brosens, Birgit Gellersen, Johanna M. T. de Vreeden-Elbertse, Cobi J. Heijnen, Nick S. Macklon

**Affiliations:** 1 Laboratory of Neuroimmunology and Developmental Origins of Disease (NIDOD), University Medical Center Utrecht, Utrecht, The Netherlands; 2 Division of Reproductive Health, Warwick Medical School, University of Warwick, Warwick, United Kingdom; 3 Endokrinologikum Hamburg, Hamburg, Germany; 4 Department of Reproductive Medicine and Gynaecology, University Medical Center Utrecht, Utrecht, The Netherlands; 5 Human Development and Health, Faculty of Medicine, University of Southampton, Princess Anne Hospital, Southampton, United Kingdom; VU University Medical Center, The Netherlands

## Abstract

**Background:**

The aetiology of recurrent miscarriage (RM) remains largely unexplained. Women with RM have a shorter time to pregnancy interval than normally fertile women, which may be due to more frequent implantation of non-viable embryos. We hypothesized that human endometrial stromal cells (H-EnSCs) of women with RM discriminate less effectively between high-and low-quality human embryos and migrate more readily towards trophoblast spheroids than H-EnSCs of normally fertile women.

**Methodology/Principal Findings:**

Monolayers of decidualized H-EnSCs were generated from endometrial biopsies of 6 women with RM and 6 fertile controls. Cell-free migration zones were created and the effect of the presence of a high-quality (day 5 blastocyst, n = 13), a low-quality (day 5 blastocyst with three pronuclei or underdeveloped embryo, n = 12) or AC-1M88 trophoblast cell line spheroid on H-ESC migratory activity was analyzed after 18 hours. In the absence of a spheroid or embryo, migration of H-EnSCs from fertile or RM women was similar. In the presence of a low-quality embryo in the zone, the migration of H-EnSCs of control women was inhibited compared to the basal migration in the absence of an embryo (P<0.05) and compared to the migration in the presence of high-quality embryo (p<0.01). Interestingly, the migratory response H-EnSCs of women with RM did not differ between high- and low-quality embryos. Furthermore, in the presence of a spheroid their migration was enhanced compared to the H-EnSCs of controls (p<0.001).

**Conclusions:**

H-EnSCs of fertile women discriminate between high- and low-quality embryos whereas H-EnSCs of women with RM fail to do so. H-EnSCs of RM women have a higher migratory response to trophoblast spheroids. Future studies will focus on the mechanisms by which low-quality embryos inhibit the migration of H-EnSCs and how this is deregulated in women with RM.

## Introduction

The relative inefficiency of human reproduction is reflected in a high prevalence of pre-implantation embryo losses, pre-clinical pregnancy losses and clinical miscarriages [1], [2]. This high rate of early loss is considered to represent a strategy for dealing with the high prevalence of chromosomal abnormalities in human embryos [3], [4].

More than ten percent of clinical pregnancies end in miscarriage [5]. Recurrent miscarriages (RM), defined as three or more consecutive miscarriages, is experienced by 1–2% of couples that try to conceive [5], [6]. Since the prevalence of RM is higher than what would be expected by probability alone, it is likely to indicate specific aetiologies in affected women. Known causes include fetal genetic abnormalities, uterine abnormalities, antiphospholipid syndrome and thrombophilic disorders. However, in more than 50% of cases, no cause is identified [5].

Increasing evidence suggests that some women may experience RM when ‘super-receptive’ endometrium allows embryos of low viability to implant, presenting as a clinical pregnancy before miscarrying [3], [7], [8].

The concept of super-receptivity is supported by the recent observation of a reduced interval between pregnancies in women with RM compared to that reported by normally fertile women [9]. Further evidence comes from studies demonstrating lower levels of endometrial mucin-1, an anti-adhesion molecule that contributes to the barrier function of the epithelium in women with RM [7]. Moreover, endometrial stromal cells (H-EnSCs) of women with RM demonstrate abnormal decidualization *in vitro* [9]. This phenotype may result in the window of implantation being extended [9], while reducing the ability of the decidualized endometrium to be ‘selective’ in response to embryo quality [10]. This concept is consistent with the previously reported association between implantation occurring later in the luteal phase and pre-clinical pregnancy loss [11].

Decidualized H-EnSCs appear to play an active role in the process of embryo implantation, demonstrating motility at the site of embryo implantation [12]. This process is regulated by Rho GTPases in the H-EnSCs [12], [13]. Moreover, decidualized H-EnSCs demonstrate invasion into Matrigel coated inserts, and the invasion of decidualized H-EnSCs increases significantly in co-culture with extra-villous trophoblast cells (AC1M88 cell line) [14].

Our aim was to investigate the migratory activity of H-EnSCs from women with RM to extravillous trophoblast cells or high- or low- quality embryos. We hypothesized that H-EnSCs from women with RM discriminate less efficiently between high- or low-quality embryos than H-EnSCs from normal fertile women. In addition, we hypothesized that ESCs from women with RM migrate more readily to extravillous trophoblast cells than H-EnSCs from normal fertile. To test these hypotheses we first determined the migratory response to extravillous trophoblast cells by comparing the *in vitro* migration of H-EnSCs obtained from women with RM and fertile controls in the presence of trophoblast spheroids. Having successfully demonstrated this, we were able to examine the migratory response of H-EnSCs from both groups in the presence of high- or low-quality human embryos.

## Results

### Participant Characteristics

Table 1 summarizes characteristics of the women with RM and normally fertile controls included in this study. No statistically significant differences in age, BMI or menstrual cycle length were observed. All women described themselves as non-smokers. As anticipated, the women in the recurrent miscarriage group reported significantly more pregnancies and spontaneous abortions than the control group. The benign conditions for which the hysterectomies were performed were uterus myomatosis (2x), vaginal bleeding disorder e.c.i. (2x), metrorrhagia (1x) and abdominal pain e.c.i. (1x).

**Table 1:** Characteristics of the women enrolled in the study.

Characteristic	RM (n = 6)	Controls (n = 6)	P-value
Age (y)^a^	33 (2)	36 (5)	0.19
Weight (kg)^a^	67(9)	69 (7)	0.72
BMI^a^	24 (3)	24 (2)	0.81
Cycle (days)^a^	28 (3)	29 (2)	0.57
Gravidity^b^	6 (4–7)	2 (1–3)	0.0001
Parity^b^	0 (0–1)	2 (1–3)	0.006
Spontaneous abortions^b^	6 (4–7)	0 (0–1)	<0.0001

a Data represent mean (SD).

b Data presented as median (range).

### Migratory Response Towards Trophoblast Spheroids

To determine whether H-EnSCs of RM women and of control fertile women differ in their migratory response to extravillous trophoblast cells and to refine our understanding of the role of different cell types in embryo-endometrial signalling, we analyzed the migratory activity of decidualized H-EnSCs of RM women and controls in response to a trophoblast spheroid based on a previously described migration assay [15].

**Figure 1:**
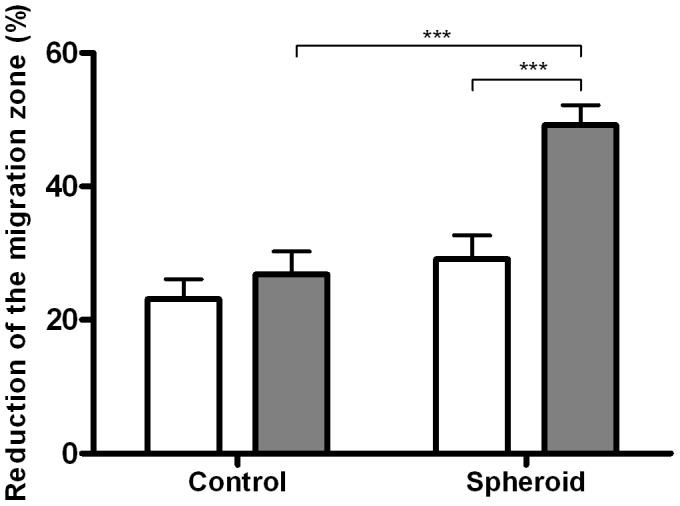
Migration of H-EnSCs of fertile control and RM women in response to a trophoblast spheroid. In a confluent well of a 48-well plate a migration zone was created. H-EnSCs were left to migrate in the presence or absence of a trophoblast spheroid in the migration zone. Data is shown as a reduction of the migration zone after 18 hours. Experiments were performed in triplicates. Data represent means ± SEM of 6 women with RM (grey bars) and 6 normally fertile women (white bars) and was analysed by 2-way ANOVA and Bonferroni post hoc tests, ***p<0.001.

In the presence of a trophoblast spheroid, the migration of decidualized H-EnSCs from women with RM was significantly more extensive compared to the decidualized H-EnSCs of normally fertile women (p<0.001) (Figure 1). The difference in migratory response seen between the control and RM group was not due to differences in spontaneous migration, as the migration in the absence of a trophoblast spheroid was comparable between the two groups (Figure 1). The pronounced migratory activity of H-EnSCs from women with RM is illustrated in the Supporting information, Video S1. This video shows an 18 hour timelapse imaging of decidualized H-EnSCs from a RM woman in the presence of a spheroid consisting of 3000 extravillous trophoblast cells. Trophoblast spheroid attachment to the culture dish, spheroid expansion and trophoblast shedding are clearly visible. Interestingly, H-EnSC migration seems to increase once the shedded trophoblast cells reach the H-EnSCs.

**Figure 2:**
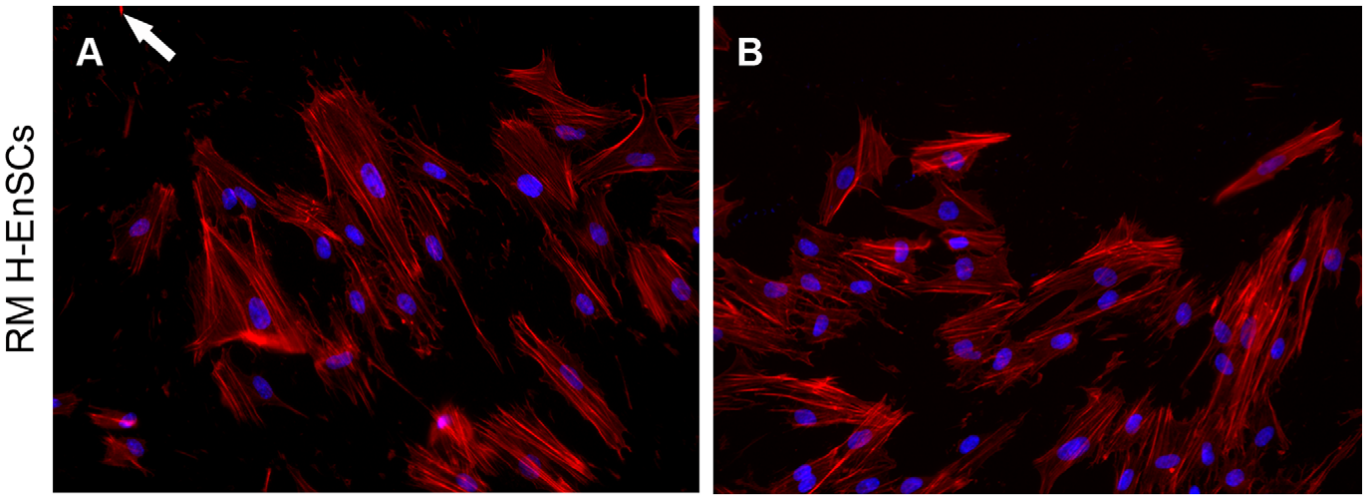
F-Actin architecture in H-EnSCs in the presence and absence of a trophoblast spheroid. In a confluent well of a 48-well plate a migration zone was created. Decidualized H-EnSCs of a RM patient were left to migrate in the presence (A) or absence (B) of a trophoblast spheroid consisting of 3000 cells. The white arrow in panel A indicates the position of the trophoblast spheroid. Both micrographs were obtained from the same location in the well. Cells were fixed and stained for F-actin (red) and DNA was stained with DAPI (blue). Magnification: ×20.

An example of cytoskeletal changes of decidualized H-EnSCs of a RM woman in the leading edge of the migration zone is depicted in Figure 2. In the presence of a spheroid in the migration zone, their actin filaments in decidualizing cells orientate to the direction of the trophoblast spheroid (Figure 2A). This directed actin filament positioning is not observed in the absence of trophoblast spheroid in the migration zone (Figure 2B).

**Figure 3:**
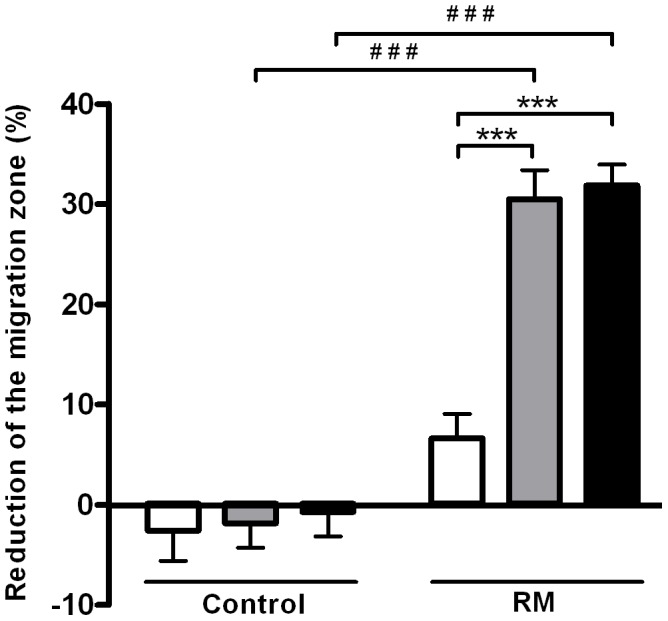
Migration of H-EnSCs in response to three different sizes of trophoblast spheroids. In a confluent well of a 48-well plate a migration zone was created. H-EnSCs were left to migrate in the presence or absence of a three different sizes of trophoblast spheroids (consisting of either 12, 40 or 120 cells as depicted by the white, grey or black bars respectively) in the migration zone. Data is shown as a reduction of the migration zone after 18 hours (the percentage reduction of the migration zone in the presence of a trophoblast spheroid minus the percentage reduction in the absence of a trophoblast spheroid). Experiments were performed in triplicates. Data represent means ± SEM of 2 women with RM and 3 normally fertile women and was analysed by 2-way ANOVA and Bonferroni post hoc tests, *** and ^###^p<0.001.

To investigate whether H-EnSC migration is dependent on the distance from the trophoblast spheroid, we split up the migration zone in five parts and compared H-EnSC migration in the central zone, adjacent to the trophoblast spheroid, to the average migratory activity of cells in the two most peripheral parts. Migration proximal and distal to the trophoblast spheroid was comparable for both decidualized H-EnSCs of women with RM and normal fertile women (p>0.05). As day 5 human embryos consist of fewer trophoblast cells than contained in the spheroids [16], we determined whether the migration of H-EnSCs of RM women is also enhanced in the presence of small trophoblast spheroids. Figure 3 shows that the migratory activity of decidualized H-EnSCs from RM patients in the presence of the small trophoblast spheroids was significantly greater than of decidualized H-EnSCs from control women (p<0.0001). Even in the presence of a 40-cell and 120-cell sized trophoblast spheroid (fewer than normally present in a day 5 human embryo), migration of H-EnSCs of women with RM was significantly enhanced compared to the basal migration (p<0.001, not shown) and compared to the migration in the presence of a 12-cell spheroid (p<0.001) (Figure 3.). In contrast, migration of H-EnSCs of normal fertile women was no longer affected by the presence of the 12-, 40- and 120-cell sized spheroids. The basal migration of decidualized H-EnSCs from women with RM did not differ significantly in comparison to the decidualized H-EnSCs from control women (p>0.05).

This data highlights the more active migratory responsiveness of decidualized H-EnSCs of RM women towards extravillous trophoblast cell signals.

### Migration in the Presence of Human Day 5 Embryos

To test whether H-EnSCs from women with RM also demonstrate increased migratory activity in the presence of a human embryo compared to H-EnSCs from fertile controls, we measured the migratory response of decidualized H-EnSCs in the presence or absence of day 5 human high-quality embryos.

Before the start of the experiment, the migration zones of H-EnSCs of women with RM and control women were comparable in size (p>0.05, data not shown). Of the 26 day-5 embryos used, three were in the morula stage M3 (embryo with signs of compaction). Of the 23 blastocysts used, two were graded as B1 (clearly expanded blastocyst, at least twice as big as the egg, with signs of hatching), 14 were graded as B2 (expanded blastocyst without signs of hatching) and 7 were graded as B3 (blastocyst with little or no expansion). All embryos used had less than 10% fragmentation and all blastocysts had an adequate number of cells in the inner cell mass and trophectoderm.

**Figure 4:**
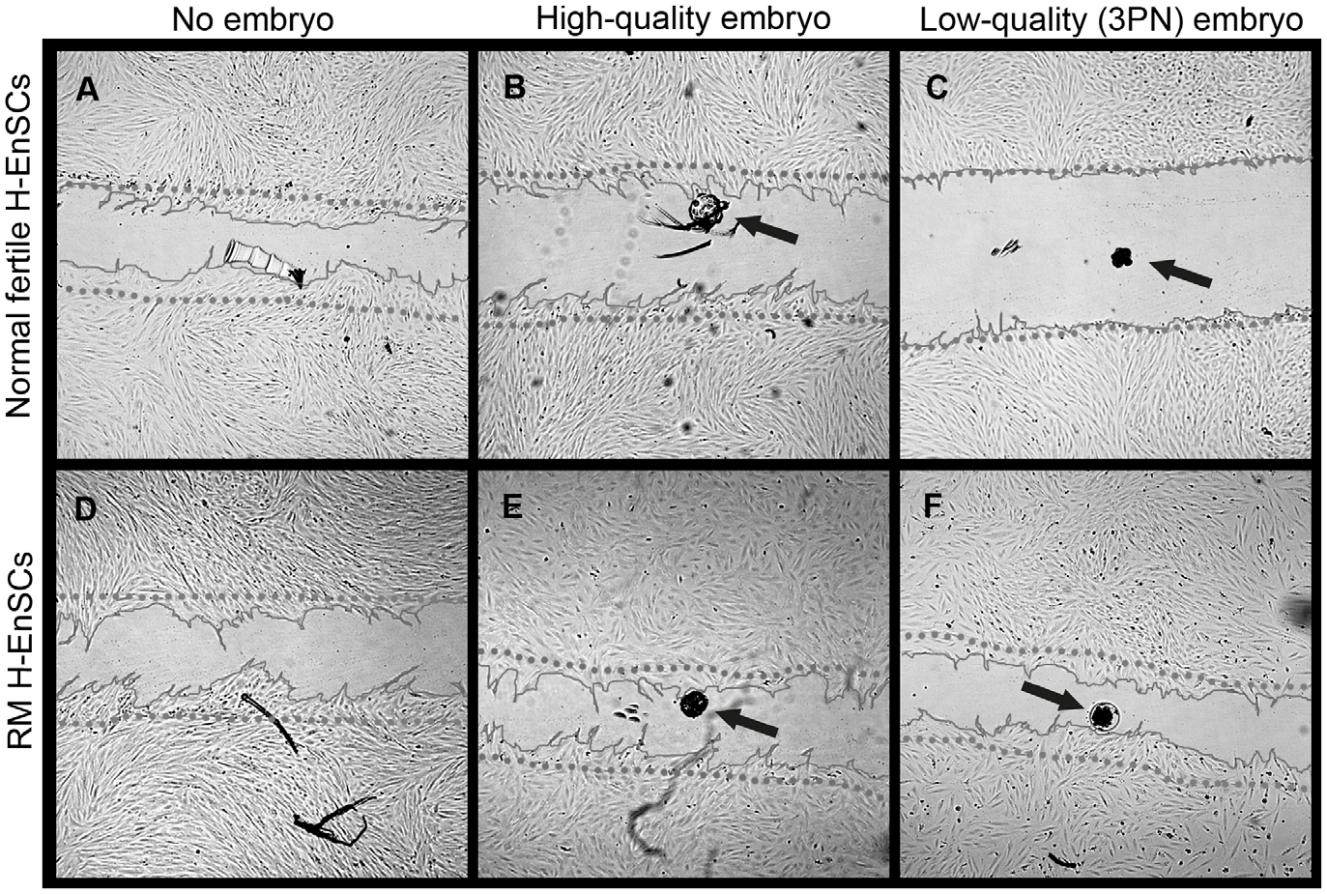
The migration zone after adding a high-quality, low-quality or no embryo. The migratory response of decidualized H-EnSCs from normally fertile (A–C) and RM women (D–F) was analyzed in absence of a human embryo (A and D), in presence of a high-quality embryo (B and E) or a low-quality embryo (C and F). Phase contrast pictures were taken 18 hours after creating the migration zone. The dotted line represents the front of the migration zone directly after its creation. As a reference for the position of the embryo, the bottom of the plate was marked. The arrows indicate the position of the embryo. All pictures were taken with 25x magnification.

**Figure 5:**
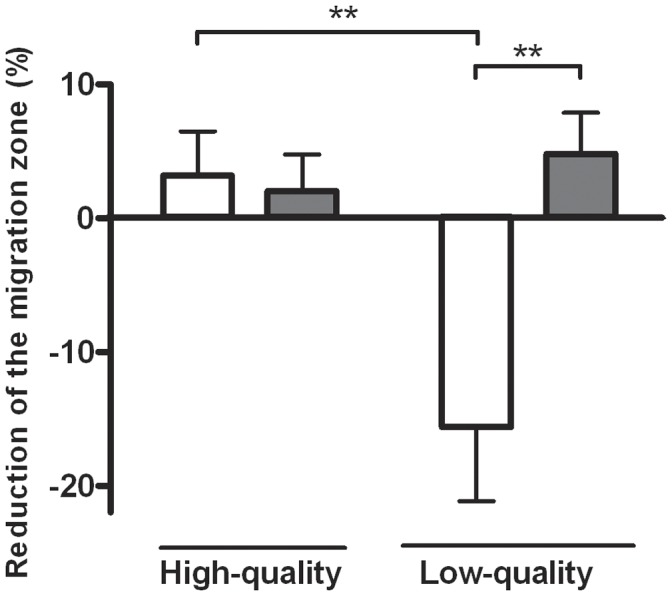
Migration of decidualized H-EnSCs in response to a high-quality or a low-quality human embryo. In a confluent well of a 4-well plate a migration zone was created. Decidualized H-EnSCs from control women (white bars) and RM women (grey bars) were left to migrate for 18 hours in the presence or absence of a high- or low-quality embryo. Data is shown as percentage reduction of the migration zone (the percentage reduction of the migration zone in the presence of an embryo minus the percentage reduction in the absence of an embryo). Data represent means ± SEM of 3 women with RM and 3 controls in the presence of a high-quality embryo (n = 13) or a low-quality embryo (n = 12) and were analysed by 2-way ANOVA and Bonferroni post hoc tests **p<0.01.

The presence of high-quality embryos did not significantly alter the migration characteristics of decidualized H-EnSCs of RM women as compared to migration in the absence of an embryo (Figure 4 and 5). Moreover, no difference in migration between decidualized H-EnSCs from fertile control women and from RM women in the presence of a high-quality embryo was observed (Figure 4 and 5).

Next, we compared the migratory response of the decidualized H-EnSCs of both groups in the presence of a low-quality embryo (Figure 4 and 5). Interestingly, in response to low-quality embryos there was a clear difference between H-EnSCs from RM and control women (p<0.01); the presence of a low-quality embryo markedly reduced migration of control H-EnSCs compared to migration in the absence of an embryo (p<0.05). In contrast, however, migration of H-EnSCs from women with RM was not reduced by the presence of a low-quality embryo compared to the migration in the absence of an embryo. The migration of H-EnSCs from women with RM was comparable in the presence of a high- or a low-quality embryo and comparable to the migration of H-EnSCs from control women in the presence of a high-quality embryo (Figure 4 and 5).

These findings indicate that H-EnSCs from RM women fail to discriminate between high- and low-quality embryos.

Video S2 of the supporting information shows timelapse recording of decidualized H-EnSCs of a woman with RM migrating towards a human 3PN blastocyst. In a period of 18 hours both H-EnSCs migration, and simultaneous growing and rolling of the 3PN embryo towards the H-EnSCs monolayer is seen. Most embryos (both 3PN and 2PN) observed, however, do not roll but remain in the migration zone. Embryo adherence to the migrating H-EnSCs is therefore rarely observed. Finally, as also became apparent from Video S2, in our experiments hatched blastocysts did not appear to adhere to the plastic dishes, which is in line with previous observations [10], [12], [15].

## Discussion

Here we show for the first time that decidualized H-EnSCs from fertile women are able to adjust their migratory activity in response to the quality of an embryo. In addition, decidualized H-EnSCs from RM women fail to discriminate between high- and low-quality embryos as the cells do not regulate their migratory response in response to the quality of the embryo. This inability to discriminate with respect to migration between low- and high-quality embryos may be regarded as a failure of nature’s quality control and is consistent with the “Selection Failure” hypothesis for women with RM [7], [8]. This hypothesis proposes that super-receptive endometrium may not be able to select high- from low-quality embryos leading to the implantation of low-quality embryos and -as their development fails- may cause a subsequent miscarriage.

The ability to distinguish between high- and low embryo qualities may have evolved as a response to the pressure of the high percentage of chromosomally abnormal (poor-quality) embryos present in fertile women [4]. A preference for the implantation of high-quality embryos may offer a reproductive advantage; higher monthly fecundity rate and larger offspring size thereby ensuring a more effective gene pool spread. A situation where the endometrial bio-sensor does not function properly may then result in the implantation of low-quality embryos which are rejected later, presenting as a clinical miscarriage.

Consistent with this concept, we have recently shown that decidualized human endometrium responds to the presence of a low-quality embryo by downregulating the production of key pro-implantation cytokines [3], [10]. Moreover, bovine endometrium gene profiles have been shown to alter in response to embryos of low versus high development potential [17].

Several studies have investigated the migratory role of H-EnSCs in regulating implantation. Decidualized H-EnSCs from fertile women display profound motility around a high-quality human blastocyst, as was illustrated by 24-hour timelapse pictures of a blastocyst co-culture with CellTracker stained H-EnSCs [12]. However, this study did not provide information about net migratory activity because the embryo was placed on top of the endometrial monolayer. Furthermore it has been shown that the migratory activity of H-EnSCs is increased in the secretory phase, since decidualized H-EnSCs of premenopausal women migrate significantly more than undifferentiated ESCs in the presence of extra-villous trophoblast secretory products [14].

In this paper we confirm the migratory nature of decidualized H-EnSCs. In addition, a series of key novel findings shed light on the mechanisms which underlie the ‘selective’ phenotype of H-EnSCs. For decidualized H-EnSCs originating from both control and RM women, migration in the presence and absence (basal migration) of a high-quality embryo is comparable. Since the presence of a high-quality embryo did not appear to result in a difference in migration compared to the basal migration observed when no embryo was present, we further hypothesized that high-quality embryos do not elicit a migratory response. The comparable basal migration and migration in the presence of a high-quality embryo is also not due to a ceiling effect as on average only 20–30% of the migration zone of the cultures with high-quality embryos was closed after 18 hours. Moreover, we observed an inhibition of the migration of normal fertile ESCs in the presence of a low-quality embryo. This implies that the distance between the embryo and the H-EnSC monolayer is not a limiting factor in regulating H-EnSC migration. It also suggests that normal fertile H-EnSCs are able to sense and respond differently to low-quality embryos.

The proposed mechanism that may underlie the ‘super-receptive’ phenotype seen in women with RM is supported by their inability to discriminate high- from low-quality embryos. This makes these RM women more receptive for low-quality embryos than normally fertile women. The ‘super-receptive’ phenotype of women with RM is also supported by the observation that these cells are more sensitive to extravillous trophoblast cell stimuli, as the migration of H-EnSCs from RM women is also enhanced by exposure to trophoblast spheroids containing fewer cells than normally present in the day 5 human embryo.

The difference in response pattern of H-EnSCs from women with RM to trophoblast spheroids and embryos is likely to reflect major differences in signalling from trophoblast spheroids and from embryos, with the former producing a stronger pro-migration signal unmodulated by signals from the other cell types represented in an embryo. It may be that H-EnSCs from women with RM are more sensitive to a stimulatory signal coming from the trophoblast spheroid or it may be that the H-EnSCs from women with RM are not able to sense an inhibitory signal coming from the trophoblast cells. In the cultures with human day 5 embryos, the failure of H-EnSCs from women with RM to respond to inhibitory signals coming from the 3PN embryo may underlie the difference between RM and fertile control women.

An interesting observation from the Supporting information, Video S1 and S2 is the intense interaction between the H-EnSCs and the trophoblast spheroid or day 5 3PN human embryo. In Video S1 it is seen that H-EnSC migration is enhanced from the moment spheroid derived satellites have reached the H-EnSC monolayer. In this case extensive directed migration towards the trophoblast spheroid is also observed, which however is not observed consistently in all spheroid migration experiments. Video S2 provides some novel insights into the earliest human embryo implantation events. It shows how a 3PN blastocyst travels to one side of the H-EnSC monolayer and rolls alongside it. It also shows the fast H-EnSC response to the 3PN blastocyst by migrating towards and away from the blastocyst, retraction and repulsion. In our experience, hatched human blastocysts do not adhere to the plastic cultures dishes, which may reflect the disposable material used. Also other groups noted a similar lack of adhesion or outgrowth when a trophoblast spheroid or embryo was placed on or adjacent to decidualized H-EnSCs [12], [15].

We have chosen to use 3PN embryos as we consider they are a suitable model for the ‘low-quality embryo’. The rate of aneuploid concepti in mid-late first trimester RM has been reported to be 30%, versus around 2% detected by chorion villus sampling of intact first trimester pregnancies [18]. Moreover, triploidy is one of the more commonly found aneuploidies in concepti examined after RM [18]. Since triploid fetuses very rarely reach term [19] there is clearly a selection mechanism in place, and this is likely to occur prior to any pregnancy becoming clinically evident [2].

Although we should acknowledge that morphology is only part of what determines embryo quality, karyotyping of some blastomeres may not reflect the rate of mosaicism. For this, CGH would have to be done in all blastomeres, which was not feasible.

The mechanisms by which healthy H-EnSCs sense the difference between high- and low-quality embryos remain to be fully elicited. Compromised embryos are metabolically very active, producing increased ATP and reactive oxygen species and demonstrating increased amino acid turnover when compared to viable embryos 20–22. Conversely, increased metabolism (as illustrated by amino acid turnover) has been shown to be associated with increased DNA damage and lower cytogenic health in the embryo [23]. These metabolites might be good candidate signals of embryo quality to be sensed by H-EnSCs. Secondly, it is known that syncytiotrophoblast shed microparticles that can subsequently reach the maternal circulation [24]. Early trophoblast cells might also shed microparticles. Decidualized H-EnSCs may then adjust their migration in response to these particles. Thirdly, human chorionic gonadotropin (hCG), a hormone secreted by the embryonic trophoblast, may modulate the response of H-EnSCs. It has been shown that decidualized H-EnSCs of RM women display a dysregulated response to bHCG with regard to prolactin and prokineticin1 mRNA expression [9]. In line with this, it is possible that decidualized H-EnSCs from women with RM also have a dysregulated migratory response to hCG. Decidualized H-EnSCs from women with RM may be less discriminatory in response to one of the products released by low-quality embryos (e.g. byproducts of increased metabolism, trophoblast microparticles or hormones such as bHCG) and as a result show no inhibition of migration.

An interesting follow-up experiment would be to stimulate the decidualized H-EnSCs with culture medium of the human embryo or trophoblast spheroid. So far, we have not been able to identify factors that are secreted by the blastocyst. Extensive crosstalk between the H-EnSCs and the trophoblast has been reported to occur at the gene expression level in a co-culture system [25].

In view of the “selection failure” hypothesis we would expect a somewhat higher live-birth rate than in RM patients that conceive naturally. However, we would not expect a dramatic increase in live birth rate following PGS for patients that suffer from RM because PGS only screens for a few number of chromosomal abnormalities, while there is a high incidence of mosaicism in young couples that undergo IVF [26] and the fecundity rate of RM is not low (with a live birth rate of around 35%). Furthermore, although the best available evidence suggests a similar live-birth rate in women with unexplained RM after PGS vs. natural conception (42 vs. 35% respectively), this is still under debate as no RCT or non-randomized comparative studies (directly comparing PGS vs. natural conception) have been performed in this study group [27].

Thus far no treatment options exist for women with unexplained recurrent miscarriage. There is insufficient evidence to support the use of immunotherapy, hCG or progesterone in women with unexplained RM to increase live birth rate.

In conclusion, we report several new findings that describe the mechanisms behind the ‘selective phenotype’ seen in fertile women and behind the ‘super-receptive’ phenotype seen in women with RM. Decidualized H-EnSCs of fertile women may actively select a high-quality embryo for implantation by inhibiting their migratory response in the presence of low-quality embryos. In contrast, the migration of H-EnSCs of RM women that is already elicited by trophoblast spheroids and by low-quality embryos highlight the super-receptive state of the endometrium of women suffering from RM. This enhanced migratory response of H-EnSCs of RM women towards a low-quality embryo or towards small spheroids may become a biomarker for identifying ‘selection failure’ as the aetiology in those patients diagnosed with otherwise unexplained RM.

Future studies will focus on the mechanisms by which low-quality embryos inhibit the migration of decidualized H-EnSCs of fertile women and how this is deregulated in the H-EnSCs of RM women. Clarification of the mechanism of non-discriminative migration of to high- and low-quality embryos of H-EnSCs of women with RM may have important clinical implications both for understanding the aetiology of this distressing condition, and for the future development of a therapeutic target for the prevention of further miscarriages.

## Materials and Methods

### Human Endometrial Stromal Cells and Surplus Embryos

This study was approved by the Medical Review Ethics Committee University Medical Center Utrecht and the Central Committee on Research involving Human Subjects in The Netherlands (NL30143.000.09). Written informed consent was obtained from all participating subjects, either for the use of surplus cryopreserved embryos or endometrial biopsies.

Day 4 embryos were thawed by taking them through consecutive washes of 1.25, 1.00, 0.75 and 0.375 mol/l DMSO for 5 minutes each, after which they were transferred to Human Tubal Fluid culture medium supplemented with 10% GPO (human plasma solution; CLB, The Netherlands) and were overlaid with 1 ml of light paraffin oil (Irvine Scientific, Santa Ana, USA), and cultured until day 5. At day 5 the embryos were scored according to previously published morphological criteria [28]. Embryos with two pronuclei (PN) on day 1 that reached the blastocyst stage with clear cavitation on day 5 were considered high-quality embryos. Day 5 blastocysts with 3PN on day 1 or those which had failed to progress beyond the morula stage were considered low-quality embryos.

H-EnSCs were isolated from endometrial biopsies obtained from six women with a history of unexplained RM (defined as three consecutive miscarriages before a gestational age of twenty weeks, with no identifiable cause) and from hysterectomy specimens of six premenopausal control women (operated for benign indications) with no history of RM. Endometrial biopsies were performed in the mid-proliferative phase of the cycle of women with RM and the biopsies of normal control women were taken randomly in the cycle. All H-EnSCs cultures were expanded and the cells frozen at −150°C in aliquots and thawed consecutively for the co-culture experiments ensuring identical co-culture conditions in all migration experiments.

### Culture Conditions and Decidualization

All 12 individual H-EnSC cultures were isolated from proliferative phase endometrium. The endometrial tissue was finely minced and enzymatically digested in 10 ml 417 U/ml collagenase type IA (Sigma) in digest medium (phenolred-free Dulbecco’s modified Eagle medium (DMEM)/F12 medium supplemented with 1% L-glutamine (Gibco), 1% amphotericin-B (Sigma, UK) and 1% penicillin/streptomycin solution (Gibco)) for 1 hour in the incubator (37°C under atmospheric oxygen levels and 5% CO2). To stop collagenase action, the digested tissue was placed in DMEM/F12 medium supplemented with 10% heat-inactivated fetal bovine serum (FCS) and pelleted by centrifugation at 670 g for 8 minutes. The cells were cultured in standard medium (digest medium supplemented with 10% FCS (Gibco) in 75 cm^2^ tissue culture flasks in the incubator. After 3 hours medium was replaced to out select the glands.

In 48-well plates 50.000 pre-decidualized H-EnSCs were seeded. In 4-well plates, 25.000 undifferentiated H-EnSCs were plated and subsequently decidualized for 5 days. Decidualization was induced by the addition of 0.5 mM of 8-bromoadenosine 3′,5′-cyclic monophosphate (8-Br-cAMP; Sigma, UK) and 1 µM medroxyprogesterone acetate (Sigma, UK) for 5 days. For this medium and for the experimental medium the FCS was replaced by 10% charcoal stripped FCS. The medium was changed every 48 hours. All primary H-EnSCs were used before passage 6.

### Trophoblast Spheroids

The AC-1M88 cell line, derived from human extravillous trophoblast cells, was cultured until 70–80% confluence, trypsinized and counted. Spheroids were formed as previously described [15]. In short, in each well 100 µl medium containing 12, 40, 120 or 3000 cells and methylcellulose was plated in a round bottom non-adherent plate. Overnight in an incubator (37°C, 5% CO2) the cells formed spheroids. One hour prior to the start of the experiment the spheroids were transferred to experimental medium.

### Migration Assay

A cell-free strip, the migration zone, was created by scratching a select area of confluent H-EnSC monolayers in the 4- and 48-well plates using a 1000 µl pipette-tip. A trophoblast spheroid, a high-quality embryo (n = 13), a low-quality embryo (n = 12), or no (controls) human embryo was placed in the migration zone. All but one H-EnSC primary cell lines were tested with two high- and two low-quality embryos. One control H-EnSC primary cell line was tested with two high-quality and three low-quality embryos. The culture medium was overlayed with mineral oil to prevent evaporation. Phase contrast pictures of the migration zone were taken using a Zeiss Axio Observer inverted microscope and the AxioVision imaging system (Zeiss, Germany) directly after creating the migration zone and 18 hours later. Using Photoshop software, the migratory response was quantified over the 18 hours.

### Staining

H-EnSC monolayers were washed in PBS and successively fixed in 4% paraformaldehyde at room temperature for 10 minutes, and permeabilized with 1% Saponin. Tetramethyl rhodamine isothiocyanate- conjugated phalloidin was used to detect filamentous actin and DAPI was used to stain DNA. Fluorescent images were obtained using an EVOS® fl digital inverted fluorescence microscope (Advanced Microscopy Group, USA).

### Statistical Analysis

Statistical testing was performed by Student’s t-tests and two-way ANOVA with Bonferroni correction. A P-value below 0.05 was considered statistically significant.

## Supporting Information

Video S1 **Timelapse of H-EnSCs of a woman with RM migrating towards a 3000 cell trophoblast spheroid.**
(MOV)

Video S2 **Timelapse of H-EnSCs of a woman with RM migrating towards a human 3PN blastocyst.**
(MOV)
